# A complicated Vogt-Koyanagi-Harada presenting with bilateral papillitis in a 5-year-old– case report

**DOI:** 10.1186/s12348-025-00481-x

**Published:** 2025-03-14

**Authors:** Salem Almerri, Raed Behbehani

**Affiliations:** Albahar Eye Center, Ibn Sina Hospital, Kuwait City, Kuwait

**Keywords:** Vogt-Koyanagi-Harada, Pediatrics, Preschool, Adalimumab, Uvietic glaucoma, Children

## Abstract

**Purpose:**

We report a case of a 5-year-old patient with Vogt-Koyanagi-Harada presenting atypically with bilateral papillitis and refractory inflammation, leading to uveitic glaucoma and necessitating an escalation of adalimumab to 40 mg biweekly.

**Observations:**

A 5-year-old girl presented with a 3-week history of eye redness, excessive lacrimation, and photophobia. Her medical history was unremarkable. On examination, her best-corrected visual acuity (BCVA) were 20/80 and 20/100 in right and left eye, respectively, with normal intraocular pressure (IOP). Anterior segment examination revealed fine keratic precipitates, anterior chamber inflammation (+ 4 cells and flare), and semi-dilated pupils with posterior synechiae. Posterior segment evaluation was limited by severe vitritis. Laboratory investigations were unremarkable except for HLA-DR4, DR52, and DR53 positivity. Optical coherence tomography (OCT) of the optic nerve showed increased retinal nerve thickness. Initial treatment with corticosteroids and methotrexate failed to achieve remission. Attempts to taper corticosteroids resulted in recurrence of anterior chamber flare, prompting the introduction of adalimumab at 20 mg/biweekly. Despite relative stability, persistent anterior chamber inflammation and subsequent corticosteroid tapering led to the development of uncontrolled uveitic glaucoma requiring surgical peripheral iridectomy. Postoperatively, adalimumab was escalated to 40 mg/biweekly, enabling successful tapering of corticosteroids. Over a 9-month follow-up period, the patient remained flare-free, with BCVA improving to 20/20 in both eyes.

**Conclusions and importance:**

This case highlights an atypical presentation of VKH in a preschool-aged child, characterized by bilateral papillitis without exudative retinal detachment. Escalation of adalimumab to 40 mg biweekly effectively controlled inflammation, facilitated corticosteroid tapering, and preserved visual acuity.

## Introduction

Vogt-Koyanagi-Harada (VKH) is a multisystem inflammatory condition that affects the eyes, central nervous system, auditory system, and integumentary system [1]. The ocular manifestations of this progressive inflammatory condition usually start with bilateral granulomatous panuveitis and serous retinal detachment followed by posterior fundus depigmentation in later stages. Systemic manifestations range from prodromal symptoms resembling viral illness to vitiligo, alopecia, headache, hearing loss, and poliosis during the chronic stage [2]. The exact pathophysiology is not well understood; however, it involves T-cell mediated autoimmune reaction against melanocyte-associated antigens involved in the forementioned systems [3]. The disease is known to affect middle-aged population, usually in second to fifth decade [4]. However, it has been infrequently reported in the pediatric age group [4–6]. In children, the disease has been reported to follow a more aggressive course with increased risks of complications, including cataract formation, glaucoma, subretinal fibrosis, and a higher risk of recurrence, compared to adults [7]. We report a case of VKH in a 5-year-old child, and highlight the challenging aspects in the management and long-term outcomes in pediatric patients.

## Case report

A 5-year-old female of Middle Eastern descent, presented with a 3-week-history of eye redness, excessive lacrimation, and photophobia. Her past medical, surgical, ophthalmic, vaccination, and family history were unremarkable.

**Fig. 1:**
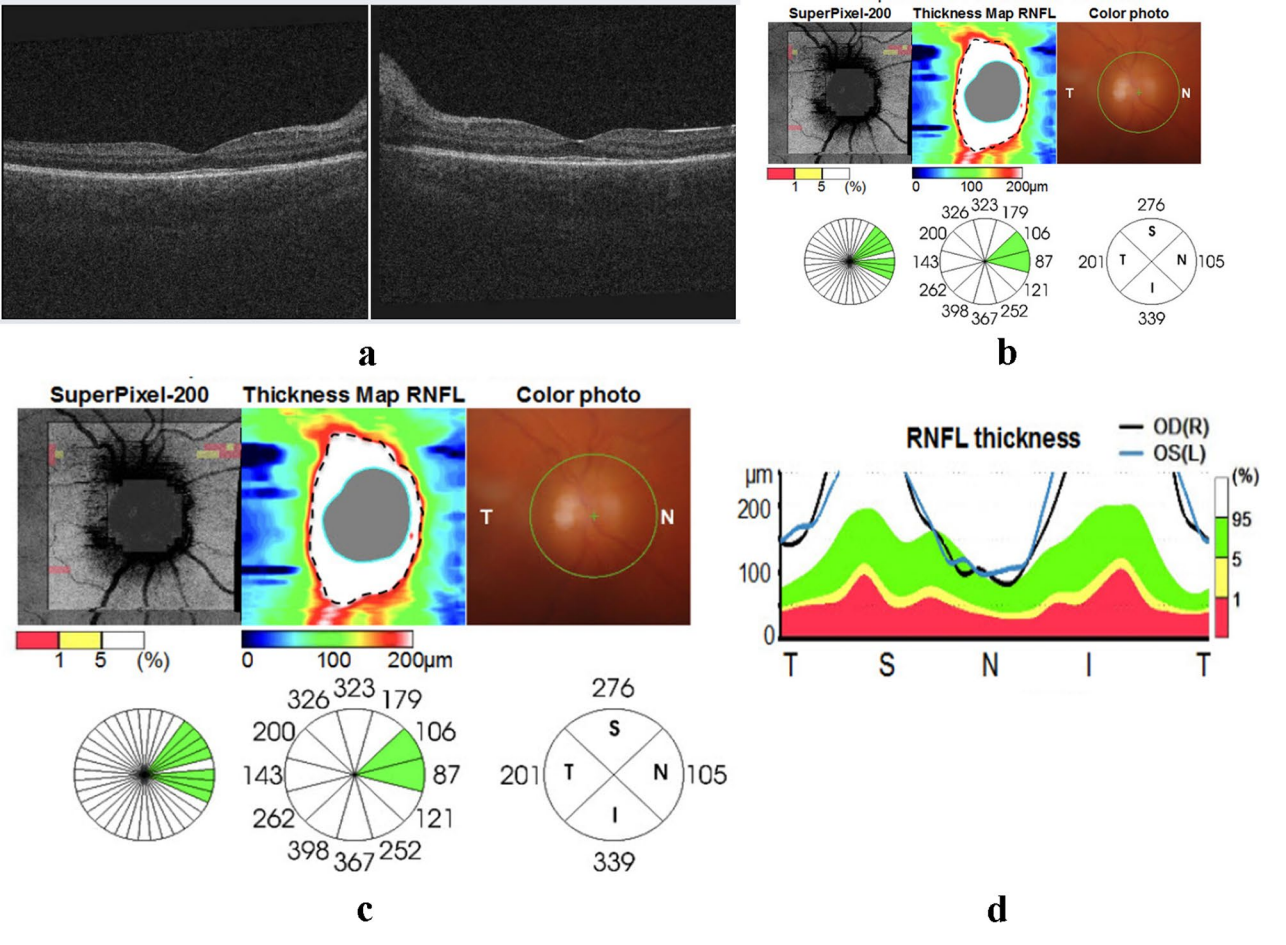
**a** Optical Coherence Tomography (OCT) scans of the macula from the right eye (right panel) and left eye (left panel) demonstrate a normal macular structure. Additionally, no abnormalities in choroidal structures, such as increased choroidal thickness or choroidal folds, are observed in these scans. **b** Optic Computed Tomography (OCT) of the right optic disc during acute uvietic phase (right) that demonstrates optic disc edema (within green circle) and increased retinal nerve fiber layer (RNFL) thickness (within the dashed line). **c** Similar findings in the in the left optic disc. OCT of left optic disc (left - within green circle) and increase in RNFL thickness (right - within the dashed line). **d)** Retinal Nerve Fiber Layer (RNFL) thickness graph that confirms the presence of RNFL thickness in both eyes

Her best-corrected visual acuity (BCVA) was 20/80 in the right eye and 20/100 in the left eye. Intraocular pressure (IOP) was 13 mmHg in the right eye and 14 mmHg in the left eye. Anterior Segment examination revealed fine keratic precipitates (KPs) and significant anterior chamber reaction graded as + 4 cells and + 4 flare bilaterally. The pupils in both eyes were semi-dilated with posterior synechiae. Fundus examination showed a severe vitritis that precluded clear view of the posterior segment. Optical coherence tomography (OCT) of the macula revealed no exudative retinal detachment, choroidal abnormalities, or macular pathology. (Figure 1a) However, OCT of the optic disc demonstrated increased retinal nerve fiber layer thickness in both eyes. (Figure 1b, c and d). The tuberculin skin test, QuantiFERON assay, chest X-ray, and Venereal Disease Research Laboratory (VDRL) test were negative. HLA typing revealed positivity for HLA-DR4, DR52, and DR53. Complete blood count (CBC) showed leukocytosis while other parameters within normal limits.

The patient was admitted and treated with intravenous methylprednisolone 500 mg/day for 5 days, hourly topical corticosteroids and cyclopentolate. Following treatment, her visual acuity improved to 20/50 in the right eye and 20/30 in the left eye, with anterior chamber inflammation reduced to + 1 cells bilaterally. She was discharged on oral prednisolone (40 mg once daily), topical prednisone, methotrexate (15 mg/weekly) and cyclopentolate. Oral prednisolone was tapered down by 10 mg every 2 weeks until reaching to 10 mg/daily in which the patient experienced a new flare of anterior uveitis, necessitating an increase in prednisolone dose back to 40 mg once daily and initiation of adalimumab (Humira) 20 mg/biweekly. Two weeks later, poliosis on eyebrows and hair started to appear. Vitiligo was also noted on her back and left arm, which was managed with topical corticosteroids. Additionally, a fundus examination revealed a sunset glow fundus and Dalen-Fuchs nodules. At this stage, incomplete VKH disease was confirmed. Over the next eight months, prednisolone dose was gradually tapered to 10 mg daily.

The patient developed uveitic glaucoma during follow-up evaluations, prompting the initiation of dorzolamide/timolol combination therapy and a trial of YAG peripheral iridotomy (PI). Six months later, the patient returned with a one-day history of severe ocular pain and photophobia. Examination revealed 360° posterior synechiae, corneal edema, conjunctival injection, iris bombe, and iris atrophy in the right eye, with an IOP of 69 mmHg. She underwent surgical peripheral iridectomy, synechiolysis, and anterior chamber washout under general anesthesia. Postoperatively, no complications were observed, and she was later discharged on oral prednisolone 40 mg/day, topical prednisone, dorzolamide/timolol drops, and methotrexate 15 mg/weekly. Adalimumab was increased to 40 mg/biweekly and prednisolone tapered down slowly to 5 mg. She was followed up for the following nine months, in which she remains stable, with no new flares, a maintained BCVA of 20/20 in both eyes, and IOP within the normal range.

## Discussion

Vogt-Koyanagi-Harada (VKH) disease predominantly affects individuals aged 20 to 50 years, however pediatric cases have been reported, expanding our understanding of its epidemiology and clinical spectrum in younger populations. Multiple studies have explored the prevalence of VKH in children. A study conducted by P. Yang in China reviewed 2,571 patients and identified 106 (4.1%) pediatric patients under 16 years of age who were diagnosed with VKH [6]. K. Tabbara reported that 13 (13.4%) of 97 VKH patients had disease onset before the age of 14 years, one of the highest documented prevalence of VKH in a pediatric cohort [4]. The onset of VKH in children appears to be more common in older children, compared to preschool-aged children (≤ 5 years). There are 12 published case reports of preschool-aged VKH patients with the addition of our case [5]. These findings suggest that VKH is rare in early childhood, with our case of a 5-year-old patient representing one of the youngest reported in the literature.

In our case, VKH was diagnosed clinically after excluding potential differential diagnoses. While no specific serological tests can definitively confirm VKH, a systematic workup is essential to rule out other etiologies of bilateral panuveitis, including tuberculosis, syphilis, and sarcoidosis [8]. In our patient, human leukocyte antigen (HLA) typing revealed positivity for HLA-DR4, HLA-DR52, and HLA-DR53, which are classically associated with VKH. There is a strong association between HLA-DR4, particularly the HLA-DRB1*04 subtype and VKH. However, the strength of this association varies across different ethnic populations [9]. Despite this correlation, the clinical utility of HLA typing remains limited due to its dependence on the prevalence of specific HLA antigens within various populations [10]. 

Ancillary diagnostic tests are critical in supporting a VKH diagnosis and were incorporated into the 2001 Revised Diagnostic Criteria for VKH (Table 1) [11]. This criterion emphasizes the importance of fluorescein angiography (FFA), indocyanine green angiography (ICGA), enhanced depth imaging optical coherence tomography (EDI-OCT), and cerebrospinal fluid analysis, especially during acute uvietic phase. In the case reported here, incomplete VKH was confirmed during the chronic phase due to the appearance of sunset glow fundus and recurrent anterior chamber inflammation along with skin changes (poliosis, vitiligo). Our evaluation relied on clinical history, physical examination, and spectral-domain OCT (SD-OCT). SD-OCT is particularly useful for detecting exudative retinal detachment (ERD) during the acute phase of VKH and for identifying macular abnormalities in the chronic phase [12]. 

**Table 1:**
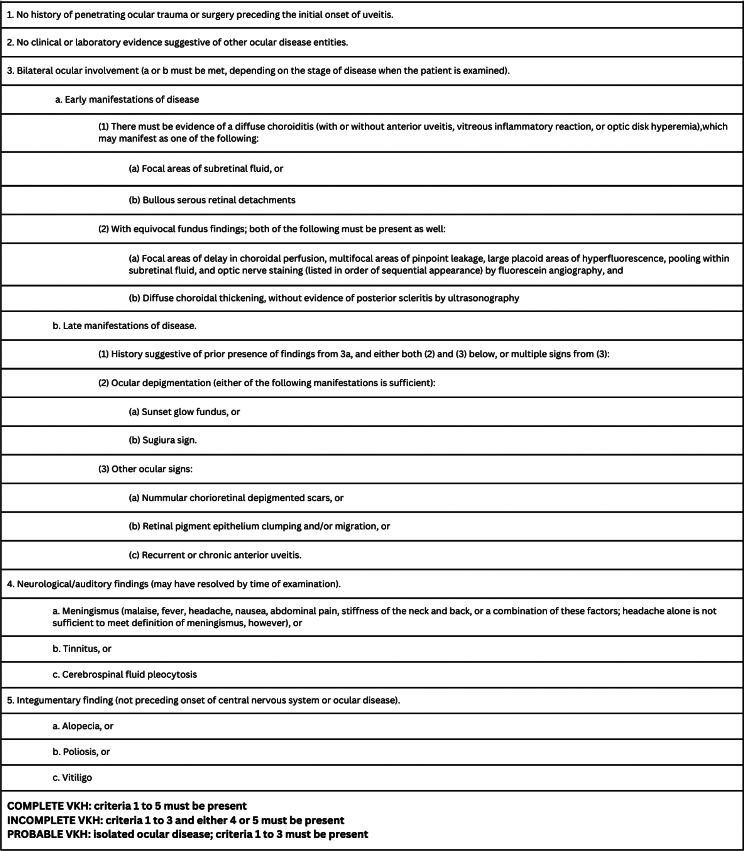
Diagnostic criteria (Revised diagnostic criteria - 2001) [11]

In this case, fundus examination and SD-OCT during the acute phase showed no evidence of ERD. While this finding is atypical, Okunuki et al. discussed the three potential subtypes of VKH: iridocyclitis, serous retinal detachment (RD), and optic disc swelling (OD). Our patient’s fundus has showed optic disc swelling on examination and OCT, raising the possibility of an OD-type VKH presentation. Okunuki et al. noted that OD-type VKH is more commonly observed in females, tends to present later in life, and is associated with a more chronic disease course compared to RD-type VKH [13]. Our patient clinical presentation aligns with the characteristics of OD-type VKH and suggests that this presentation can occur in younger VKH patients. Notably, subclinical choroiditis is more effectively detected in its early stages of VKH by ICGA, making it superior to FFA and EDI-OCT [12]. However, the forementioned imaging modalities were not utilized in the evaluation of our patient. Optical Coherence Tomography (OCT) of the Retinal Nerve Fiber Layer (RNFL) has revealed evidence of papillitis, characterized by a significant increase in RNFL thickness. According to one study, this finding is observed in approximately one-third of pediatric uveitis cases [14]. 

The management of pediatric VKH poses significant challenges due to the difficulty in controlling inflammation and the potential adverse effects of corticosteroids in children, including growth retardation and cushingoid features [15]. The standard treatment approach for VKH includes corticosteroids, immunomodulatory agents, and biologic therapies. Given the severity of bilateral panuveitis, our patient was concurrently initiated on oral corticosteroids and methotrexate. Despite initial clinical improvement, a flare-up occurred within two months of discharge following corticosteroid tapering, necessitating the increase of steroid dose. Rapid corticosteroid tapering over the duration of less than six months have been associated with increased risk of disease recurrence [3]. One study demonstrated higher recurrence rates in patients receiving systemic corticosteroids for less than six months compared to those treated for over six months [16]. 

In our case, methotrexate alone proved inadequate for corticosteroid tapering, leading to the initiation of adalimumab (Humira 20 mg/biweekly), a therapeutic option has been reported to be effective in pediatric VKH cases [17]. Adalimumab, an FDA-approved anti-TNF agent for uveitis, is a fully humanized IgG1 monoclonal antibody that neutralizes tumor necrosis factor (TNF)-alpha activity and induces apoptosis of TNF-expressing cells [18]. AlQahtani reported successful use of adalimumab (20 mg/biweekly) in a case of a. 4-year-old refractory pediatric VKH, with significant improvement of visual outcome and resolution of inflammation [19]. In our patient, adalimumab 20 mg/biweekly was unsuccessful in inducing remission of inflammation and prevention of ocular complications of uveitis. An increase in adalimumab dosage (40 mg/biweekly) was sufficient to control the inflammation, maintain a visual acuity of 20/20 and allow the tapering of corticosteroids. This case demonstrates the efficacy of adalimumab in the management of refractory pediatric VKH, especially in preschool population.

Young children with VKH are at an increased risk of developing complications during the recurrent chronic phase compared to adults, mainly cataracts and glaucoma [3]. The etiology of uveitic glaucoma is multifactorial, includes prolonged corticosteroid use and angle closure resulting from posterior synechiae or peripheral anterior synechiae formation due to recurrent inflammation [20]. In our case, the patient had multiple episodes of acute intraocular pressure (IOP) elevation, which did not respond initially to antiglaucoma medications and laser iridectomy. Surgical peripheral iridectomy was ultimately required to treat the persistent IOP elevation, along with the elevation of adalimumab dose.

In conclusion, VKH can present in pre-school children with mainly optic disc edema and, in this case, adalimumab at a dose of 40 mg/biweekly effectively controlled inflammation and facilitated slow corticosteroid tapering. Further studies are needed to investigate the use of adalimumab in preschool VKH patients and to define its optimal dosing and long-term efficacy.

## Data Availability

No datasets were generated or analysed during the current study.
